# Effects of *ChondroT* on potassium Oxonate-induced Hyperuricemic mice: downregulation of xanthine oxidase and urate transporter 1

**DOI:** 10.1186/s12906-018-2415-2

**Published:** 2019-01-08

**Authors:** Dool-Ri Oh, Jong Ro Kim, Chul Yung Choi, Chan-hun Choi, Chang-su Na, Bok Yun Kang, Seon-Jong Kim, Young Ran Kim

**Affiliations:** 1 College of Pharmacy and Research Institute of Drug Development, Chonnam National University, Gwangju, 61186 Republic of Korea; 2 Jeonnam Bioindustry Foundation, Jeollanamdo Institute of Natural Resources Research (JINR), Jeonnam, 59338 Republic of Korea; 3 MEGA BIO Co., Ltd, Jeonnam, 58141 Republic of Korea; 4 College of Korean Medicine, Dongshin University, 185 Geonjae-ro, Naju-si, Jeollanam-do 58245 Republic of Korea; 5 Department of Korean Medicine Rehabilitation, Mokpo Oriental Hospital of Dongshin University, 313 Baengnyeon-daero, Mokpo, 530-822 South Korea

**Keywords:** *ChondroT*, Hyperuricemia, URAT1, Uric acid, Xanthine oxidase

## Abstract

**Background:**

*ChondroT*, a new herbal medication, consists of the water extracts of *Osterici* Radix, *Lonicerae* Folium, *Angelicae Gigantis* Radix, *Clematidis* Radix, and *Phellodendri* Cortex (6:4:4:4:3). We previously reported that *ChondroT* showed significant anti-arthritis and anti-inflammatory effects.

**Methods:**

This study was designed to evaluate the effect of *ChondroT* on hyperuricemia. First, the effect of *ChondroT* was evaluated on xanthine oxidase (XOD) activity in vitro. The anti-hyperuricemic effect of *ChondroT* was also studied in potassium oxonate (PO)-induced hyperuricemic model mice. Uric acid (UA) and XOD were evaluated in the serum, urine, and liver of the mice. In addition, we measured serum creatinine (Cr) and blood urea nitrogen (BUN) levels as well as mRNA expression of the mouse urate transporter 1 (mURAT1) to evaluate kidney function and urate excretion in hyperuricemic mice.

**Results:**

*ChondroT* showed in vitro XOD inhibitory activity in a dose-dependent manner (*P* < 0.05). We demonstrated that *ChondroT* (37.5, 75 and 150 mg/kg) significantly reduced serum UA (*P* < 0.01 and *P* < 0.001, respectively), and upregulated urinary UA (*P* < 0.001, respectively) in PO-induced hyperuricemic mice. In addition, *ChondroT* (75 and 150 mg/kg) significantly reduced Cr (*P* < 0.05 and *P* < 0.01, respectively), BUN (*P* < 0.05 and *P* < 0.001, respectively), GOT (*P* < 0.05 and *P* < 0.01, respectively), and GPT (*P >* 0.05 and *P* < 0.05, respectively) levels in PO-induced hyperuricemic mice. *ChondroT* (75 and 150 mg/kg) also significantly downregulated serum (*P* < 0.05) and liver (*P* < 0.05) XOD activity. Compared to the hyperuricemic mice, the *ChondroT* (37.5, 75, and 150 mg/kg)-treated mice showed decreased mURAT1 protein expression level.

**Conclusion:**

*ChondroT* displayed anti-hyperuricemic effects by regulating XOD activity and kidney mURAT1.

## Background

Hyperuricemia is characterized by an increase in blood uric acid (UA) levels [1]. UA is the last metabolite in the purine catabolic pathway formed by the enzyme xanthine oxidase (XOD). The high blood UA levels cause the accumulation of urate crystals in the joints, which is known as an important risk factor for gouty arthritis, UA nephrolithiasis, and kidney disease [2]. In humans, 90% of the filtered urate is reabsorbed, which is mediated by specific transporter molecule such as urate transporter 1 (URAT1). It functions as the major mechanism for regulating blood urate levels. It has also been reported that a defect in human URAT1 (hURAT1) is associated with the pathogenesis of hyperuricemia and gout [3, 4]. Thus, XOD and URAT1 are important targets to regulate hyperuricemia and gout. Potassium oxonate-induced hyperuricemic mice are appropriate experimental models for investigating the mechanism underlying hyperuricemia and for developing therapeutic agents. Allopurinol (AP) is an anti-hyperuricemic agent that acts by reducing XOD activity and serum UA levels. However, allopurinol may cause serious side effects such as skin rashes, allergic reactions [5], and gastrointestinal toxicity [6]. Therefore, there is an obvious need for novel agents for the physiological regulation of UA levels and prevention of hyperuricemia.

Gangwhaljetongyeum (GHJTY), a traditional Korean herbal medicine composed of 18 herbs, has been used to treat severe joint pain, limited motion, fever, and swelling [7]. We previously reported that GHJTY inhibited the inflammatory processes associated with arthritis [8]. Using bioinformatics analysis [9] and screening experiments, we selected the following five effective herbal constituents of GHJTY, *Osterici* Radix*, Lonicerae* Folium, *Angelicae Gigantis* Radix, *Clematidis* Radix, and *Phellodendri* Cortex and named the resulting concoction as *ChondroT* [10]. *ChondroT* showed more significant multifunctional therapeutic effects on inflammation and arthritis than GHJTY did [10]. Some reports have shown the strong correlation between serum urate levels and generalized osteoarthritis [11, 12]. UA elevations may be evaluated as a major risk factor for osteoarthritis tissue damage, osteoarthritis progression, or both through inflammasome activation [13, 14]. In the present study, we investigated the effects of *ChondroT* on in vitro and in vivo the xanthine oxidase activity as well as its anti-hyperuricemia effects in PO-induced hyperuricemic mice.

## Methods

### Materials and reagents

The XOD, xanthine, allopurinol (AP), and PO were purchased from Sigma-Aldrich (St. Louis, MO, USA). The UA and XOD assay kits were purchased from Cayman Chemical (Ann Arbor, MI, USA) and Biovision (Mountain View, CA, USA), respectively.

*ChondroT* was prepared using a method previously described [10]. The five herbal medicines forming *ChondroT* were purchased from Omniherb (Yeongcheon, Korea). Briefly, the five herbal constituents, *Osterici* Radix (Korea), *Lonicerae* Folium (China), *Angelicae Gigantis* Radix (Korea), *Clematidis* Radix (China), and *Phellodendri* Cortex (China) were combined in a ratio of 6:4:4:4:3 (Table 1). Next, each five herbal constituents as well as *ChondroT* extracted once using a 10-fold volume of water as the solvent at 100 °C for 3 h. After filtration (180-mesh), the water extract was concentrated using a continuous vacuum evaporator (at approximately 55–60 °C, 670 mmHg), followed by lyophilization using a vacuum drier (720 mmHg) for 8 h. The extraction yield of *ChondroT* was about 29.5%. The resultant *ChondroT* and each herbal constituents was dissolved in phosphate-buffered saline (PBS) and filter-sterilized. For phytochemical analysis for *ChondroT* extract used in this study, a convenient and accurate HPLC–PDA detection was previously conducted for simultaneous determination of seven reference components, chlorogenic acid, berberine Cl, nodakenin, isoferulic acid, oxypeucedanin hydrate, decursin, and decursinol angelate [10].

**Table 1:** Composition of ChondroT and the used parts of 5 herbs

Latin name	Scientific name	Used part	*w*/w %
Angelicae Gigantis Radix	*Angelica gigas* Nakai	root	19.0
Clematidis Radix	*Clematis mandshurica* Ruprecht	root	19.0
Lonicerae Folium	*Lonicera japonica* Thunberg	leaf	19.0
Osterici Radix	*Ostericum koreanum* Maximowicz	root	28.6
Phellodendri Cortex	*Phellodendron amurense* Ruprecht	tree bark	14.3

### Evaluation of in vitro XOD inhibitory activity

XOD activity were measured by modified procedure protocol of Wang et al. (2008) [15]. *ChondroT* at concentrations of 100, 300, and 500 μg/mL was added to 0.1 M potassium phosphate buffer (pH 7.5) containing 0.4 U/mL XOD and then incubated for 10 min at 37 °C. The xanthine substrate (0.4 mM) was added to the test solutions, which were incubated for 10 min at 37 °C, and then the absorbance of each sample was measured at 295 nm. AP was used as a positive control at a final concentration of 1.36 μg/ml. The XOD inhibitory activity was expressed as the percentage inhibition of XOD in the assay mixture system.

### Experimental mice and treatments

Five week old male ICR mice weighing 25–30 g were purchased from Orient Bio (Seongnam, Republic of Korea). The specific-pathogen free (SPF) mice were randomly divided into 6 groups (*N* = 5). The animals were maintained at a constant room temperature of 22 ± 2 °C with a humidity of 50 ± 5% and were allowed free access to water and food under a 12-h light/dark cycle (lights on at 8:00 a.m.). All the animal procedures were carried out in accordance with the guidelines of the Animal Care and Use Committee of Chonnam National University. The animal study was approved by Animal Care and Use Committee of Chonnam National University (Permission number: CNN IACUC-YB-2016-12). After the experiment, all animals were euthanized using Carbon dioxide (CO_2_) in accordance with the Institutional Animal Care and Use Committee (IACUC) guidelines.

### Experimental model of hyperuricemia in mice and drug administration

The hyperuricemic animal model was established by injecting the animals with PO, a urate oxidase inhibitor [16, 17]. PO were administrated according to previously described method [18], with modifications. Briefly, the mice were divided into six groups of five mice each, and *ChondroT* was orally pre-administered at doses of 37.5, 75, and 150 mg/kg/day for 7 days before the PO administration. And then, all the mice except those in the normal control group were intraperitoneally injected once daily with PO (300 mg/kg) at 10:00 a.m. for 7 days experiment. The PO was dissolved in 0.9% saline solution before use. *ChondroT* (37.5, 75, and 150 mg/kg) and AP (5 mg/kg) were orally administration at 11:00 a.m. for 7 consecutive days on the day when the PO was given. Two hours after the final drug administration, all animals were anesthetized using 2.5% isoflurane. Blood samples were collected from the abdominal aorta of the mice and then the samples were centrifuged at 3000 *g* for 10 min. The supernatant serum and urine samples were both collected and stored at − 20 °C until they were assayed. The mouse kidneys and livers were collected, washed with 0.9% cold saline solution, and stored at − 80 °C until they were assayed.

### Biochemical analysis of hyperuricemic mice

The serum sample was assayed to determine the levels of UA, creatinine (Cr), blood urea nitrogen (BUN), glucose, glutamic oxaloacetic transaminase (GOT), and glutamic pyruvic transaminase (GPT) using appropriate kits (DRI-CHEM 4000i, FUGI-FILM, Tokyo, Japan) [19, 20]. The levels of UA were measured in the collected urine samples using Cayman Chemical assay kits (Cayman, Ann Arbor, MI, USA). The serum and liver XOD activities were determined using a fluorometric assay kit (Biovision, Mountain View, CA, USA) in accordance with the manufacturer’s protocol.

### Reverse transcription-polymerase chain reaction (RT-PCR)

Total RNA was isolated from the mouse kidney cortex tissue using an easy-BLUE total RNA extraction kit (iNtRON Biotechnology, Seongnam, Republic of Korea) according to the manufacturer’s instructions. The final total RNA pellet was resuspended in 20 μL of diethyl pyrocarbonate-treated water. To synthesize the cDNA, 1 μg total RNA was mixed with a premix containing the oligo (dT) primer and diethyl pyrocarbonate-treated water in a final volume of 20 μL and incubated at 45 °C for 60 min. The reaction was stopped by heat inactivation at 95 °C for 5 min. Subsequently, the cDNA was amplified with gene-specific primers using the polymerase chain reaction (PCR) PreMix (Bioneer, Daejeon, Republic of Korea) according to the manufacturer’s instructions. The specific primers used in this study are shown in Table 2. RT-PCR analysis were prepared according to previously described method [19, 20], with modifications. Briefly, 5 μL of cDNA, 1 μL of forward primer (10 pm), 1 μL of reverse primer (10 pm), 13 μL of diethyl pyrocarbonate-treated water were added to PCR PreMix tube on ice. Thirty cycles of amplification were performed with denaturation at 95 °C for 30 s, annealing at 58 °C for 40 s, and extension at 72 °C for 50 s. All reactions were finished with a single extra cycle at 72 °C for ten minutes. The PCR products were electrophoresed on a 1.5% (*w/v*) agarose gel, stained with ethidium bromide, and then the expression levels were quantified using a gel documentation and analysis system (Gel Doc 2000, Bio-Rad, Sydney, Australia). The relative expression levels of the target genes were normalized to a glyceraldehyde 3-phosphate dehydrogenase (GAPDH) internal control.

**Table 2:** Primers and expected sizes of polymerase chain reaction (PCR) products with each primer pair

Gene	Primer sequence (5′ → 3′)	Product size (bp)	GenBank accession number
*mURAT1*	Forward: GCTACCAGAATCGGCACGCT Reverse: CACCGGGAAGTCCACAATCC	342	NM_009203
*GAPDH*	Forward: AGATCCACAACGGATACATT Reverse: TCCCTCAAGATTGTCAGCAA	309	NM_008084

### Statistical analysis

The data are presented as the mean ± standard error (S.E.) and were statistically evaluated using one-way analysis of variance (ANOVA) using the GraphPad Prism (GraphPad Inc., San Diego, California, USA) software program. The results were considered statistical significance at *P* < 0.05.

## Results

### Effects of *ChondroT* on in vitro XOD inhibitory activity

*ChondroT* at concentrations of 100, 300, and 500 μg/mL inhibited XOD activity in a dose-dependent manner by 26.94 ± 1.80%, 46.11 ± 5.26%, and 65.04 ± 7.41%, respectively, with a half maximal inhibitory concentration (IC_50_) of 414.09 μg/mL. The positive control, AP, which is an established XOD inhibitor showed an inhibition of 95.53 ± 0.13% at a concentration of 1.36 μg/mL and IC_50_ of 0.21 μg/mL (Table 3).

**Table 3:** Effects of ChondroT on in vitro xanthine oxidase (XOD) activity

Drug	Concentration (μg/mL)	Inhibition (% Mean ± S.E.)
Angelicae Gigantis Radix	100	2.82 ± 3.84
Clematidis Radix	100	11.67 ± 1.03
Lonicerae Folium	100	22.10 ± 3.72
Osterici Radix	100	8.62 ± 1.43
Phellodendri Cortex	100	19.01 ± 2.41
ChondroT	100	26.94 ± 1.80 ^*^
	300	46.11 ± 5.26 ^*^
	500	65.04 ± 7.41 ^*^
Allopurinol	1.36	95.53 ± 0.13 ^***^

Values are mean ± standard error (S.E., *n* = 3). ^*^P < 0.05 and ^***^P < 0.001 compared with negative control group

### Effects of *ChondroT* on serum and urine UA in PO-induced hyperuricemic mice

**Fig. 1:**
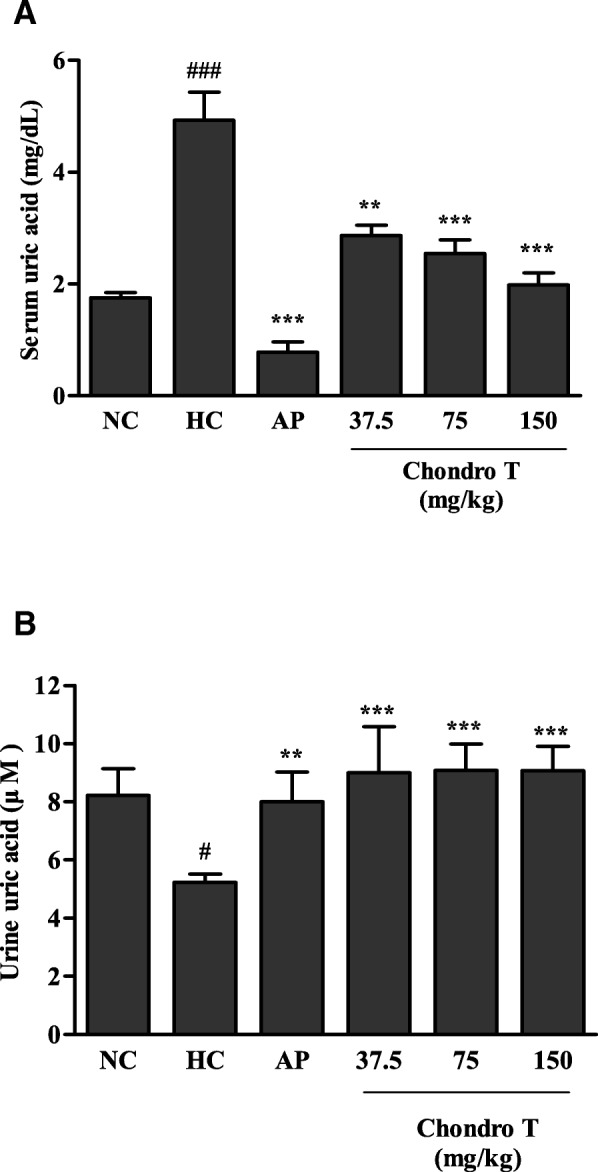
Effects of *ChondroT* on uric acid in (**a**) serum and (**b**) urine of potassium oxonate (PO)-induced hyperuricemic mice NC, normal control; HC, hyperuricemic control with potassium oxonate (300 mg/kg); AP, allopurinol (5 mg/kg). Values are expressed as the mean ± standard error (S.E., *n* = 5). ^#^*P* < 0.05 and ^###^*P* < 0.001 compared with NC group; **P < 0.01, and ***P < 0.001 compared with HC group

The anti-hyperuricemic effects of *ChondroT* were evaluated in PO-induced hyperuricemic mice by determining the UA and XOD levels in the serum, urine, and liver. Five week old male ICR mice (25–30 g) were randomly divided into 6 groups (*N* = 5). The serum UA significantly increased in the PO-induced hyperuricemic mice compared to that in the negative control (NC) mice (Fig. 1a). AP (5 mg/kg) reduced the serum UA levels of the hyperuricemic group more significantly than that of the NC group. Treatment with *ChondroT* (37.5, 75, and 150 mg/kg) decreased the serum UA in hyperuricemic mice (*P* < 0.01 or *P* < 0.001, Fig. 1a). In addition, AP (5 mg/kg) and *ChondroT* (37.5, 75, and 150 mg/kg) effectively increased the urinary level of UA (*P* < 0.01 or *P* < 0.001, Fig. 1b), suggesting an increased excretion of UA from blood into urine. These data suggest that *ChondroT* may enhance kidney urate excretion in hyperuricemic mice.

### Effects of *ChondroT* on XOD activity in hyperuricemic mice

**Fig. 2:**
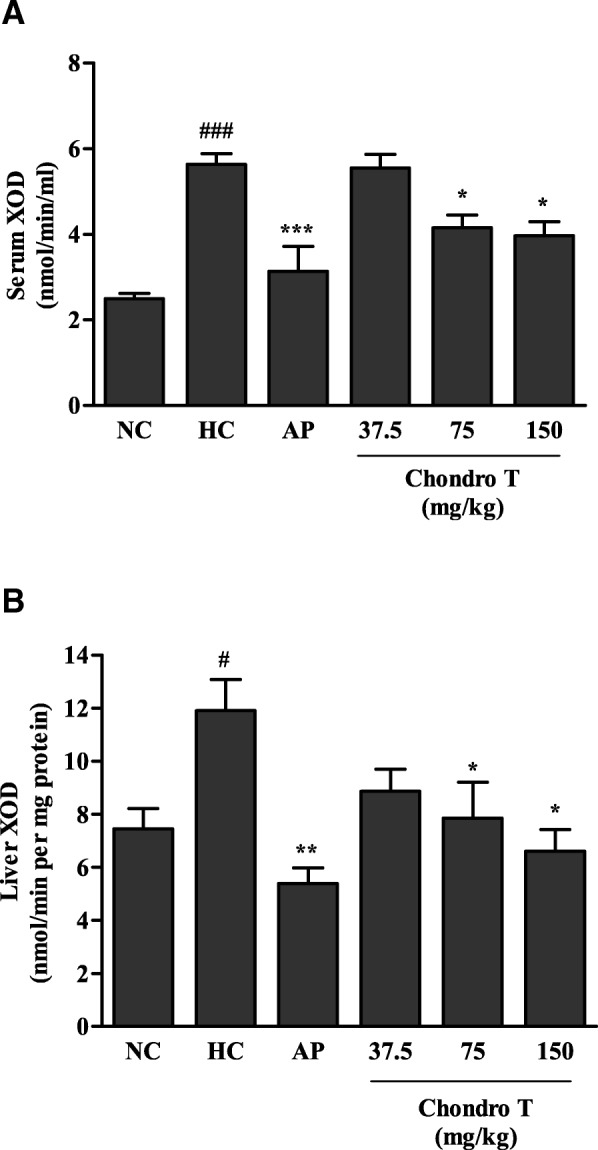
Effects of *ChondroT* on (**a**) serum and (**b**) liver xanthine oxidase (XOD) activities of potassium oxonate (PO)-induced hyperuricemic mice NC, normal control; HC, hyperuricemic control with potassium oxonate (300 mg/kg); AP, allopurinol (5 mg/kg). Values are expressed as the mean ± standard error (S.E., n = 5). ^#^*P* < 0.05 and ^###^*P* < 0.001 compared with NC group; ^*^P < 0.05, ^**^P < 0.01, and ^***^P < 0.001 compared with HC group

The effects of *ChondroT* on in vivo XOD activity were evaluated in PO-induced hyperuricemic mice. Figure 2 shows the effects of *ChondroT* and AP on serum and liver XOD activity in hyperuricemic mice. AP significantly suppressed the serum and liver XOD activity in hyperuricemic mice (*P* < 0.001 or *P* < 0.01, respectively). *ChondroT* (75 and 150 mg/kg) significantly inhibited the serum XOD activity (*P* < 0.05, Fig. 2a). We observed a similar inhibition of the liver XOD activity by *ChondroT* (75 and 150 mg/kg, *P* < 0.05, Fig. 2b).

### Effects of *ChondroT* on liver and kidney function of hyperuricemic mice

The serum biochemical parameters of each group are shown in Table 4. To evaluate the protective effects of *ChondroT* on kidney toxicity in the hyperuricemic mice, serum toxicological markers indicative of kidney injury were measured at the end of the experimental period. The serum levels of Cr and BUN significantly increased in the PO-induced hyperuricemic mice compared with that in the NC group, while treatment with *ChondroT* (75 and 150 mg/kg) decreased the levels. The *ChondroT* (37.5, 75, and 150 mg/kg) also significantly reduced the serum BUN levels in hyperuricemic mice. Furthermore, *ChondroT* alleviated the liver damage by lowering the levels of GOT and GPT, two common markers of liver damage, which were in turn increased by the PO treatment.

**Table 4:** Effects of ChondroT on serum levels of Creatinine (Cr), blood urea nitrogen (BUN), glutamic oxaloacetic transaminase (GOT) and glutamic pyruvic transaminase (GPT) in hyperuricemic mice

Blood parameter	NC	HC	AP (mg/kg)	ChondroT (mg/kg)		
			5	37.5	75	150
Creatinine (mg/dL)	0.20 ± 0.02	0.75 ± 0.04 ^###^	0.23 ± 0.01 ^**^	0.50 ± 0.04	0.28 ± 0.02 ^*^	0.23 ± 0.02 ^**^
BUN (mg/dL)	23.62 ± 0.56	40.10 ± 1.21 ^###^	21.45 ± 0.96 ^***^	26.27 ± 0.58 ^*^	22.95 ± 1.06 ^*^	20.43 ± 0.74 ^***^
GOT (U/L)	48.67 ± 0.90	73.33 ± 3.95 ^##^	39.80 ± 0.92 ^***^	54.83 ± 1.90 ^*^	46.00 ± 2.01 ^*^	39.75 ± 2.56 ^**^
GPT (U/L)	15.00 ± 1.58	28.00 ± 1.46	13.50 ± 0.91 ^*^	24.17 ± 0.95	20.33 ± 1.29	15.00 ± 1.39 ^*^

*NC*, normal control; *HC*, hyperuricemic control with potassium oxonate (300 mg/kg); AP, allopurinol (5 mg/kg). Values are mean ± standard error (S.E., n = 5). ^##^*P* < 0.01 and ^###^*P* < 0.001 compared with NC group; ^*^P < 0.05, ^**^P < 0.01, and ^***^P < 0.001 compared with HC group

### Effects of *ChondroT* on mRNA expression of mURAT1 in hyperuricemic mice

**Fig. 3:**
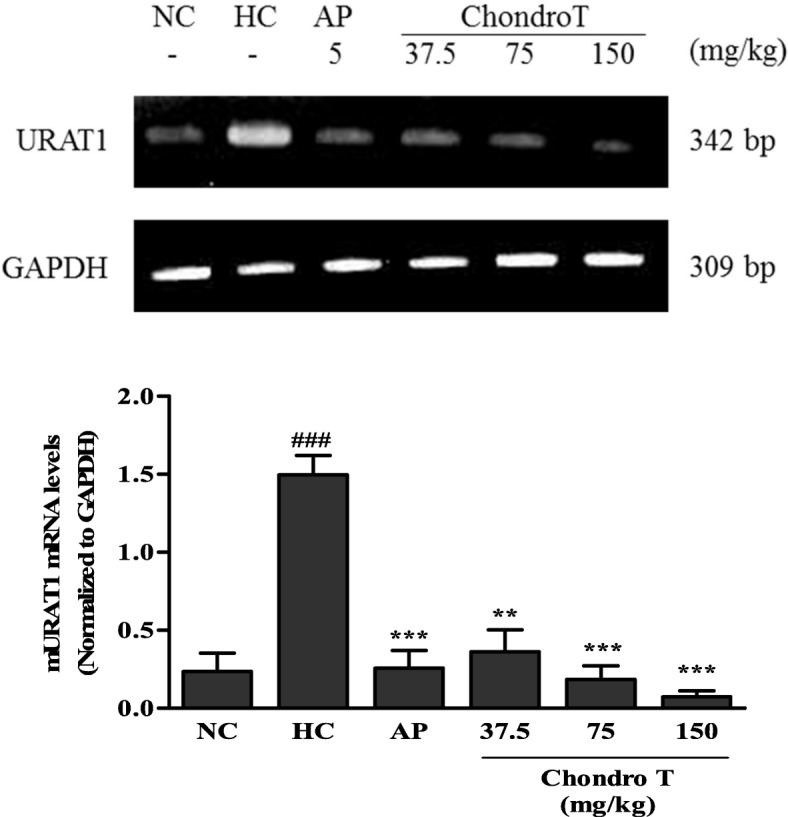
Effects of *ChondroT* on mRNA levels of mouse urate transporter 1 (mURAT1) in kidney tissues of potassium oxonate (PO)-induced hyperuricemic mice using reverse transcription-polymerase chain reaction (RT-PCR). The relative mRNA expression levels of the mURAT1 gene were normalized against GAPDH NC, normal control; HC, hyperuricemic control with potassium oxonate (300 mg/kg); AP, allopurinol (5 mg/kg). Values are expressed as the mean ± standard error (S.E., *n* = 5). ^###^*P* < 0.001 compared with NC group; ^**^P < 0.01 and ^***^P < 0.001 compared with HC group

URAT1 is an important gene involved in the transport of UA from the kidney to the blood. To confirm the mechanism underlying the effects of *ChondroT* on hyperuricemia, the URAT1 gene expression levels in the kidney tissue were investigated (Fig. 3). Compared with the NC group, the hyperuricemic control (HC) group exhibited increased URAT1 mRNA levels in the kidney tissue. However, the URAT1 mRNA expression levels of the *ChondroT* (37.5, 75 and 150 mg/kg, *P* < 0.01 and *P* < 0.001)-treated groups significantly decreased compared with that of the HC group (Fig. 3). These data suggest that *ChondroT* may enhance kidney urate excretion through the actions of URAT1 in hyperuricemic mice.

## Discussion

In the present study, we demonstrated that *ChondroT*, a new complex herbal medication reduced serum UA in hyperuricemic mice by downregulating XOD activity and renal mURAT1. Because XOD and URAT1 are important targets to regulate hyperuricemia and gout, potassium oxonate-induced hyperuricemic mice were used as appropriate experimental models for investigating the mechanism underlying hyperuricemia. XOD plays an important role in the catabolism of purines. The XOD enzyme catalyzes the oxidation of hypoxanthine to xanthine and can further catalyze the oxidation of xanthine to UA. AP, an XOD inhibitor has been used as an anti-hyperuricemia agent and anti-gout [21]. In addition, febuxostat is a novel orally administered anti-hyperuricemic drug [22] that reduces the production of UA in the body by XOD inhibition [22, 23]. We demonstrated that *ChondroT* inhibited XOD activity in vitro (Table 3). PO-treated mice can serve as a useful animal model for investigating hyperuricemia pathology. The intraperitoneal administration of 300 mg/kg PO for 7 days to mice increased the serum UA, decreased the urinary UA, and elevated the serum and liver XOD activity (Figs. 1 and 2). *ChondroT* significantly reduced the serum UA level, whereas it increased the urinary UA level in hyperuricemic mice (Fig. 1). Moreover, the *ChondroT*-treated mice showed a significant decreased in serum and liver XOD activity compared with that of the hyperuricemic mice (Fig. 2). Thus, the in vivo enzyme inhibitory activity of *ChondroT* is the net inhibitory activity of both XOD and xanthine dehydrogenase (XDH).

Hyperuricemia is frequently observed in patients with chronic kidney disease and can be accompanied by an increase in serum Cr and BUN [24]. Previous other studies reported that AP significantly blocked renal functional changes in PO-induced hyperuricemic rats and lowered Cr levels as well as UA in patients [25, 26]. We demonstrated that *ChondroT* significantly reduced the serum Cr and BUN levels in hyperuricemic mice (Table 4). Furthermore, liver dysfunction, poor blood flow in the liver and raised uric acid may cause hyperuricemia [27]. In this study, PO-induced hyperuricemic mice increased liver damages by increasing the levels of two markers for liver damages, GOT, and GPT, which was attenuated by *ChondroT* and AP.

Richette et al. [28] reported that more than 90% of all cases of hyperuricemia are caused by the impaired renal excretion of UA. Renal mURAT1, a urate transporter gene contributes significantly to the reabsorption of UA in humans. Furthermore, it is a drug target that is inhibited by uricosuric drugs such as benzbromarone and probenecid [28]. In this study, the PO-induced hyperuricemic mice showed increased expression of mURAT1 mRNA, which was significantly attenuated by *ChondroT* treatment (Fig. 3). These results suggest that *ChondroT* likely plays a role in reducing urate reabsorption with the subsequent enhancement of urate excretion in hyperuricemic mice, mainly by the downregulation of mURAT1. However, the uricosuric effect of *ChondroT* could have limited clinical use due to safety issues such as risk of uric acid stone formation. Recently, Sun et al. [11] and Acheson et al. [12] reported the involvement of serum urate levels in osteoarthritis. The association between hyperuricemia and osteoarthritis might be explained by a common risk factor rather than any direct effect of serum urate on the joints. Thus, both studies found a significant relationship between the pathogenesis of osteoarthritis and serum UA levels.

In a previous study, we demonstrated that *ChondroT* triggered significant anti-inflammatory and anti-arthritis effects [10]. Previously, we evaluated the toxicity of *ChondroT* at high doses such as 500, 1000, and 2000 mg/kg for 4 weeks at Korea Testing & Research Institute (KTR). *ChondroT* was confirmed to be safe herb at the high doses. AST and ALT levels of *ChondroT*-treated rats are normal value ranges. In addition, BUN levels in *ChondroT*-treated rats are in normal scores. *ChondroT* did not show any toxicity to liver, kidney, heart, lung, and spleen in individual histopathologic evaluations (data not shown). In this present study, *ChondroT* displayed anti-hyperuricemic effects by regulating XOD activity and kidney mURAT1. *ChondroT* is a water extract of five herbs, *Osterici* Radix*, Lonicerae* Folium, *Angelicae Gigantis* Radix, *Clematidis* Radix, and *Phellodendri* Cortex. We confirmed that of the five herbs included in the extract, *Lonicerae* Folium and *Phellodendri* Cortex strongly inhibited XOD activity in vitro (Table 3). Furthermore, *Phellodendri* Cortex has been reported to decrease serum UA and liver XOD activity in PO-induced hyperuricemic mice [29]. In addition, *Phellodendri* Cortex was reported to protect human osteoarthritic cartilage and chondrocytes [30]. Moreover, *Lonicerae* Folium and *Clematidis* Radix showed inhibitory effects against XOD activity in vitro*,* but these effects were not strong [31]. *Clematidis* Radix also has been reported to show anti-rheumatic arthritis effects that are mediated through decrease PLA_2_ activities and PGE_2_ production and lipopolysaccharide-induced COX-2 protein expression [32]. *ChondroT* showed a more significant in vivo than in vitro inhibition of XOD (Table 3 and Fig. 2). In addition, *ChondroT* showed a higher synergistic effect than each of its five herbal constituents did on XOD (Table 3). These results suggest that *ChondroT* may be a promising therapeutic candidate for the treatment of hyperuricemia-related diseases.

In our previous results, seven reference components in *ChondroT*, such as chlorogenic acid, berberine Cl, nodakenin, isoferulic acid, oxypeucedanin hydrate, decursin, and decursinol angelate were selected for quality control of *ChondroT* [10]. Chlorogenic acid has been shown to exert anti-gout activity as well as improvement on hyperuricemia and inflammation by inhibiting the XOD activity, serum UA levels, and production of proinflammatory cytokines (e.g. IL-1β and IL-6) [33]. In addition, alkaloid compounds including berberine Cl have been shown inhibition activity of XOD enzymes to reduce UA levels [29, 34]. However, further studies are required to determine the functional active constituents that are involved in the anti-hyperuricemia as well as anti-gout activity.

## Conclusion

These results showed that *ChondroT* regulates hyperuricemia by downregulating mURAT1 and inhibiting XOD enzyme. In addition, the suppressive effect on XOD enzyme in vivo was greater than that exhibited in vitro, suggesting that *ChondroT*, a new complex herbal medication, has therapeutic potential for the treatment of patients suffering from hyperuricemia and gout.

## Abbreviations

AP: allopurinol
BUN: blood urea nitrogen
Cr: creatinine
GAPDH: glyceraldehyde-3-phosphate dehydrogenase
GHJTY: Ganghwaljetongyeum
GOT: glutamic oxaloacetic transaminase
GPT: glutamic pyruvic transaminase
PO: potassium oxonate
RT-PCR: reverse transcription-polymerase chain reaction
UA: uric acid
URAT1: urate transporter 1
XOD: xanthine oxidase

## References

[1] Haidari F, Rashidi MR, Keshavarz SA, Mahboob SA, Eshraghian MR, Shahi MM (2008). Effects of onion on serum uric acid levels and hepatic xanthine dehydrogenase/xanthine oxidase activities in hyperuricemic rats. Pak J Biol Sci.

[2] Chen JHCS, Chen HJ, Yeh WT, Pan WH (2009). Serum uric acid level as an independent risk factor for all-cause, cardiovascular, and ischemic stroke mortality: a Chinese cohort study. Arthritis Rheum.

[3] Graessler J, Graessler A, Unger S, Kopprasch S, Tausche AK, Kuhlisch E, Schroeder HE (2006). Association of the human urate transporter 1 with reduced renal uric acid excretion and hyperuricemia in a German Caucasian population. Arthritis Rheum.

[4] Vazquez-Mellado J, Jimenez-Vaca AL, Cuevas-Covarrubias S, Alvarado-Romano V, Pozo-Molina G, Burgos-Vargas R (2007). Molecular analysis of the SLC22A12 (URAT1) gene in patients with primary gout. Rheumatology.

[5] Umamaheswari M, Asokkumar K, Sivashanmugam AT, Remyaraju A, Subhadradevi V, Ravi TK (2009). In vitro xanthine oxidase inhibitory activity of the fractions of Erythrina stricta Roxb. J Ethnopharmacol.

[6] Cronstein BN, Terkeltaub R (2006). The inflammatory process of gout and its treatment. Arthritis Res Ther.

[7] Kim YH, Lee JH (2001). CheongKangEuiGam.

[8] Jeoung BR, Lee KD, Na CS, Kim YE, Kim B, Kim YR (2013). Ganghwaljetongyeum, an anti-arthritic remedy, attenuates synoviocyte proliferation and reduces the production of proinflammatory mediators in macrophages: the therapeutic effect of GHJTY on rheumatoid arthritis. BMC Complemen Altern Med.

[9] Choi W, Choi CH, Kim YR, Kim SJ, Na CS, Lee H (2016). HerDing: herb recommendation system to treat diseases using genes and chemicals. Database (Oxford).

[10] Park JU, Kim SJ, Na CS, Choi CH, Seo CS, Son JK, Kang BY, Kim YR (2016). Chondroprotective and anti-inflammatory effects of ChondroT, a new complex herbal medication. BMC Complem Altern Med.

[11] Sun Y, Brenner H, Sauerland S, Gunther KP, Puhl W, Sturmer T (2000). Serum uric acid and patterns of radiographic osteoarthritis--the Ulm osteoarthritis study. Scand J Rheumatol.

[12] Acheson RM, Collart AB (1975). New Haven survey of joint diseases. XVII. Relationship between some systemic characteristics and osteoarthrosis in a general population. Ann Rheum Dis.

[13] Denoble AEHK, Stabler TV, Kelly SJ, Hershfield MS, McDaniel GE, Coleman RE, Kraus VB (2011). Uric acid is a danger signal of increasing risk for osteoarthritis through inflammasome activation. Proc Natl Acad Sci U S A.

[14] Nowatzky J, Howard R, Pillinger MH, Krasnokutsky S (2010). The role of uric acid and other crystals in osteoarthritis. Curr Rheumatol Rep.

[15] Wang SY, Yang CW, Liao JW, Zhen WW, Chu FH, Chang ST (2008). Essential oil from leaves of Cinnamomum osmophloeum acts as a xanthine oxidase inhibitor and reduces the serum uric acid levels in oxonate-induced mice. Phytomedicine.

[16] Stavric B, Clayman S, Gradd RE, Hebert D (1975). Some in vivo effects in the rat induced by chlorprothixene and potassium oxonate. Pharmacol Res Commun.

[17] Hall IH, Scoville JP, Reynolds DJ, Simlot R, Duncan P (1990). Substituted cyclic imides as potential anti-gout agents. Life Sci.

[18] Wang X, Wang C, Hu Q, Lv Y, Zhang X, Ouyang Z, Kong L (2010). The dual actions of Sanmiao wan as a hypouricemic agent: Down-regulation of hepatic XOD and renal mURAT1 in hyperuricemic mice. J Ethnopharmacol.

[19] Mohammadi A, Mirzaei F, Jamshidi M, Yari R, Pak S, Norouzian P, Abdolkarimi V, Oshaghi EA. Influence of flaxseed on lipid profiles and expression of LXRα, in intestine of diabetic rat. Int J Biol. 2013;5(4):23-8.

[20] Mohammadi A, Vafaei SA, Moradi MN, Ahmadi M, Pourjafar M, Oshaghi EA (2015). Combination of ezetimibe and garlic reduces serum lipids and intestinal Niemann-pick C1-like 1 expression more effectively in Hypercholesterolemic mice. Avicenna J Med Biochem.

[21] Elion GB, Kovensky A, Hitchings GH (1966). Metabolic studies of allopurinol, an inhibitor of xanthine oxidase. Biochem Pharmacol.

[22] Takano Y, Hase-Aoki K, Horiuchi H, Zhao L, Kasahara Y, Kondo S, Becker MA (2005). Selectivity of febuxostat, a novel non-purine inhibitor of xanthine oxidase/xanthine dehydrogenase. Life Sci.

[23] Bisht M, Bist SS (2011). Febuxostat: a novel agent for management of hyperuricemia in gout. Indian J Pharm Sci.

[24] Hoffmann D, Fuchs TC, Henzler T, Matheis KA, Herget T, Dekant W, Hewitt P, Mally A (2010). Evaluation of a urinary kidney biomarker panel in rat models of acute and subchronic nephrotoxicity. Toxicology.

[25] Kang DH, Nakagawa T, Feng L, Watanabe S, Han L, Mazzali M, Truong L, Harris R, Johnson RJ (2002). A role for uric acid in the progression of renal disease. J Am Soci Nephrol.

[26] Siu YP, Leung KT, Tong MK, Kwan TH (2006). Use of allopurinol in slowing the progression of renal disease through its ability to lower serum uric acid level. Am J Kidney Dis.

[27] Tomita M, Mizuno S, Yamanaka H, Hosoda Y, Sakuma K, Matuoka Y, Odaka M, Yamaguchi M, Yosida H, Morisawa H (2000). Does hyperuricemia aeffect mortality? A prospective cohort study of japanese male workers. J Epidemiol.

[28] Richette P, Bardin T (2010). Gout. Lancet.

[29] Kong LD, Yang C, Ge F, Wang HD, Guo YS (2004). A Chinese herbal medicine Ermiao wan reduces serum uric acid level and inhibits liver xanthine dehydrogenase and xanthine oxidase in mice. J Ethnopharmacol.

[30] Kim JH, Huh JE, Baek YH, Lee JD, Choi DY, Park DS (2011). Effect of Phellodendron amurense in protecting human osteoarthritic cartilage and chondrocytes. J Ethnopharmacol.

[31] Shin YJ, Hwang JM, Lee SC (2013). Antioxidant and xanthine oxidase inhibitory activities of hot water extracts of medicinal herbs. J Korean Soc Food Sci Nutr.

[32] Lee JS, Kim KH, Lee SD, Kim KS (2012). The effect of *Clematidis Radix* herbal-acupuncture solution, on collagen, adjuvant, lipopolysaccharide and phospholipase A2 induced rheumatoid arthritis in mice. J Korean Acupunct Moxibustion Society.

[33] Meng ZQ, Tang ZH, Yan YX, Guo CR, Cao L, Ding G, Huang WZ, Wang ZZ, Wang KD, Xiao W (2014). Study on the anti-gout activity of chlorogenic acid: improvement on hyperuricemia and gouty inflammation. Am J Chin Med.

[34] Ahmad I, Ijaz F, Fatima I, Ahmad N, Chen S, Afza N, Malik A (2010). Xanthine oxidase/tyrosinase inhibiting, antioxidant, and antifungal oxindole alkaloids from Isatis costata. Pharm Biol.

